# Flexible planning of corrective responses for double-step reduction in the number of potential targets

**DOI:** 10.1038/s41598-021-86325-9

**Published:** 2021-03-25

**Authors:** Ryoji Onagawa, Kazutoshi Kudo

**Affiliations:** 1 Laboratory of Sports Sciences, Department of Life Sciences, Graduate School of Arts and Sciences, The University of Tokyo, Tokyo, Japan; 2 Research Fellow of Japan Society for the Promotion of Science, Tokyo, Japan; 3 Interfaculty Initiative in Information Studies, Graduate School of Interdisciplinary Information Studies, The University of Tokyo, Tokyo, Japan

**Keywords:** Motor control, Sensorimotor processing

## Abstract

Humans are often required to plan/execute movements in the presence of multiple motor targets simultaneously. Under such situations, it is widely confirmed that humans frequently initiate movements towards the weighted average direction of distinct motor plans toward each potential target. However, in situations where the potential targets change in a step-by-step manner, the strategy to proceed towards the weighted average direction at each time could be sub-optimal in light of the costs of the corrective response. Herein, we tested the sensorimotor strategy followed during a step-by-step reduction of potential goals. To test the hypothesis, we compared the corrective responses when the number of targets went from three to two, and when the number of targets went from three to one at the same time. As the results, weak corrections were confirmed when the number of targets was reduced from three to two. Moreover, the corrective responses when the number of targets went from three to two was smaller than the average behavior estimated from the corrective responses when the number of targets went from three to one at the same time. This pattern of corrective responses reflects the suppression of unnecessary corrections that generate noise and cost to the control system. These results suggest that the corrective responses are flexibly modulated depending on the necessity, and cannot be explained by weighted average behavior.

## Introduction

Humans are often required to launch a movement towards multiple potential goals. In daily life, external states change continuously over time, such as during walking through a crowded space, driving a car down a town street, or making decisions in many ball sports. In such situations, humans are required to deal with external, environmental changes in the number of potential targets multiple-times and to set control strategies about when and how to correct an ongoing movement, according to each update of the potential targets. However, it has been unclear how humans correct their in-flight movement according to step-by-step decreases in the number of potential targets.

Previous studies have investigated human sensorimotor control for multiple potential reach targets using the “go-before-you-know paradigm”^1–10^. In this paradigm, an individual is simultaneously presented with multiple potential targets and is required to launch a movement before knowing the final target location, which is presented after movement onset^1–3,10^ or after passing through a given spatial threshold^4,6,8^. In such task settings, revealing the target becomes a direct trigger for correcting an ongoing movement. Moreover, many studies have found that humans frequently initiate their movements towards the weighted average direction of potential targets^1–3,5,6,10,11^. However, it was reported that this behavior is abated when its strategic benefits are decreased^4,7–9^. Specifically, if motor planning and execution for a step-by-step target revealing follows the weighted average behavior, as observed in previous studies, an individual would have to correct their in-flight movement according to changes in potential targets. However, in such situations, correcting an ongoing movement every time that the number of potential goals is decreased can be a suboptimal control strategy, since the correction of in-flight movement demands costs and incorporates noise into the sensorimotor system^11^. The parameters of the control policy that governs how sensory feedback will be used in real-time to generate motor commands are selected to minimize such cost, typically defined as a combination of energy expenditure and inaccuracy^11–14^. Considering that, the corrective response for the first change in target information could be abated. Thus, testing how correct an ongoing movement is at each phase of target information updates (i.e., decreases in the number of potential targets) could be effective for clarifying the sensorimotor control policy under uncertainty about task goals.

Besides, the distance between potential targets could modulate the strategy of corrective movements for each target update, since the benefit of the corrective movements becomes higher as the distance of potential targets becomes larger. If the distance is short, the individual could attain the final goal with the corrective response after the final target position is revealed. On the contrary, if the distance is long, such behavior has many temporal and movement-related costs. Therefore, the relative magnitude of the corrective response for in-flight movements executed in each target revision phase should be affected by the distance between potential targets.

Herein, we tested motor planning and execution in a situation where the number of potential final goals was decreased step-by-step manner. The following two conditions have been set for the target revealing process, that is double-step trials and single-step trials. In the double-step trials, there were three potential targets at the movement onset, which were reduced one by one in two steps of thresholds (1 cm and 12 cm from the start position), until finally one target was left. In the single-step trials, there were three potential targets at the beginning of the operation, and one final target was left at the first threshold (1 cm from the start position). We hypothesized that the correction of ongoing movements after the first threshold is diminished to reduce the expected correction costs through a whole movement in a given trial, and that differences in target-separation angles modulate the magnitude of the corrective response. To directly examine deviations from the averaging behavior of the motor plan for each potential target, we compared the corrective response observed in the double-step trials, and that estimated by the two averaging behavior models based on the single-step trials.

## Results

**Figure 1:**
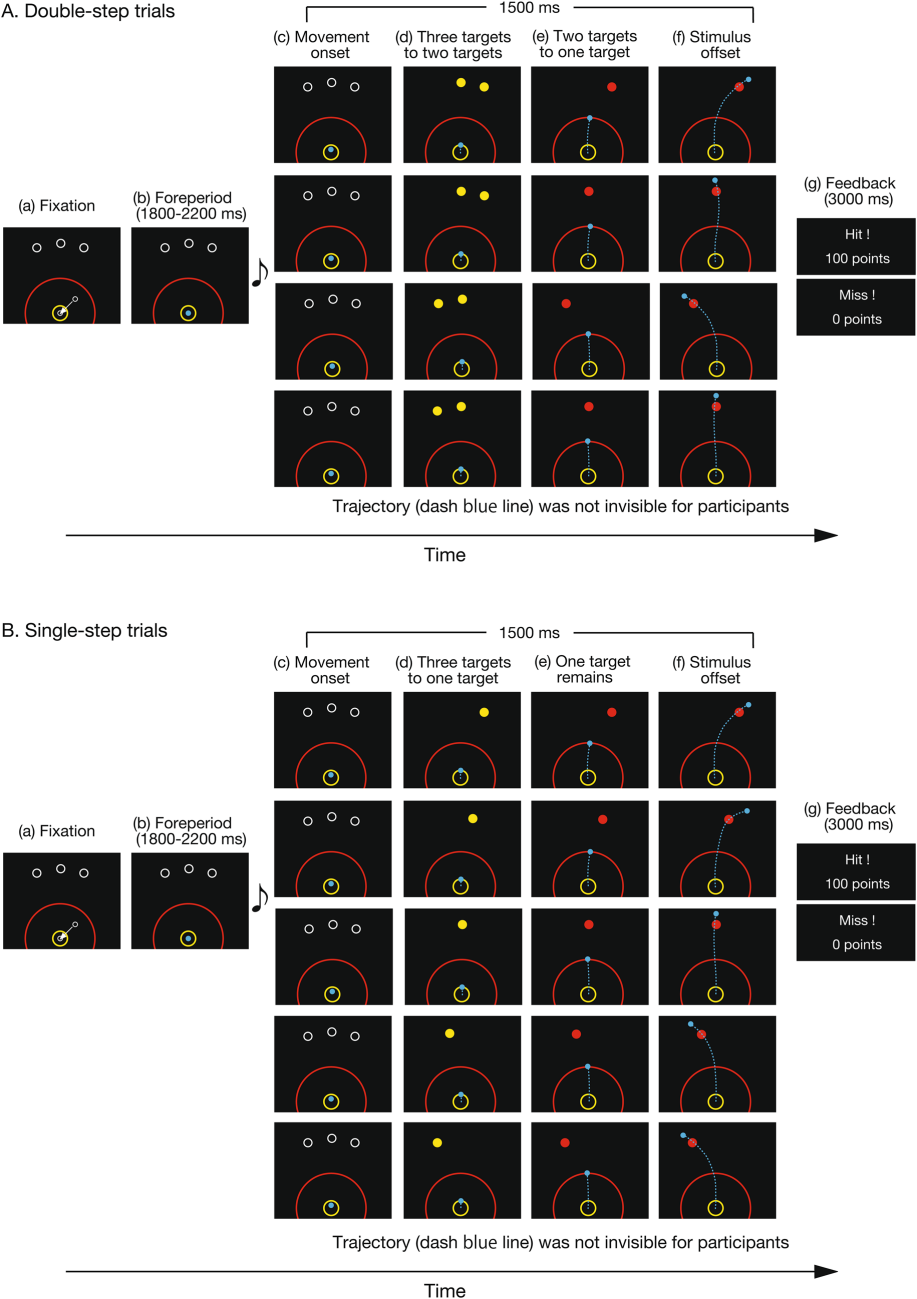
Experimental paradigm. Participants (*N* = 14) performed the double-step target-revealing task, a modified version of the go-before-you-know paradigm. The two upper rows show the right condition, the first is the condition where the final target is the right target and the second is the condition where the final target is the Center target. The two lower rows show the left condition, the first is the condition where the final target is the left target, and the second is the condition where the final target is the center target. The participants were required to move a cursor towards the final correct target. (**A**) Trial sequence in the double-step trials. To initiate the task, the participants fixated the cursor on the start position (a). At this position, the cursor turned to a blue-filled circle from a white frame circle. After a random fore-period (b), a beep sound notified the participants to initiate a movement (c). When the cursor moved 2 cm away from the start position (first threshold, yellow frame circles), one of the side potential targets disappeared, and the remaining targets turned to yellow-filled circles (d). When the cursor was moved 12 cm from the start position (second threshold, red frame circles), the final correct target turned to a red-filled circle, and the remaining target disappeared (e). After 1500 ms from movement onset, the stimuli disappeared (f), and the result of the movement was presented as feedback (g). (**B**) Trial sequence in the single-step trials. The sequence of tasks in the single-step trials is almost the same as in the double-step trials. In the order from top to bottom, the following conditions for target change are shown: right condition, medium right condition, center condition, medium left condition, and left condition. In the single-step trials, a final target was determined when the cursor moved 2 cm away from the start position (first threshold, yellow frame circles). These trials were set up to estimate corrective responses in accordance with the averaging behavior.

**Figure 2:**
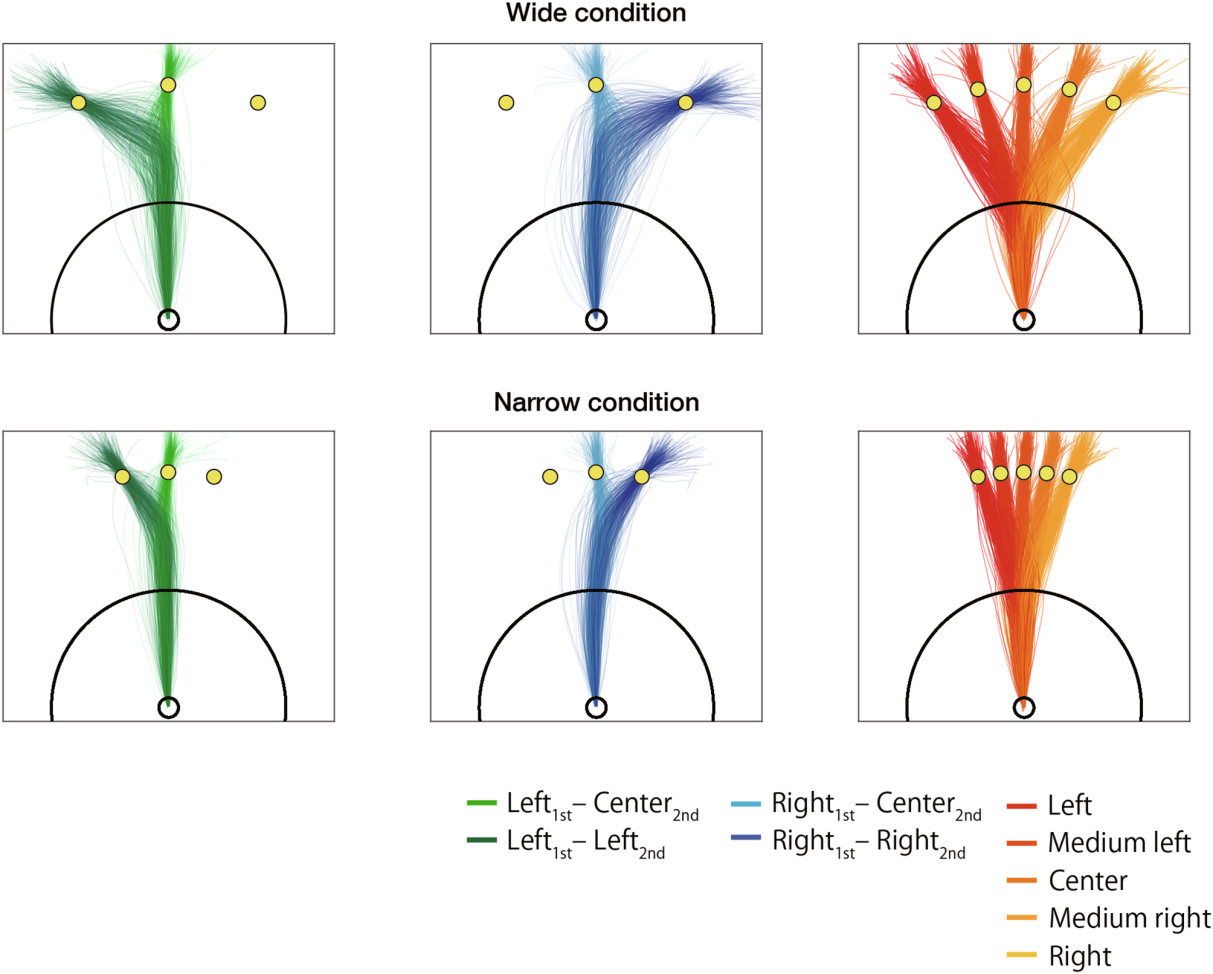
Cursor trajectories including all participants’ data. The left and center columns show cursor trajectories in double-step trials. The green (left column) and blue (right column) trajectories show the left and right conditions, respectively. The dark and light trajectories indicate the condition at which the final target was the side target and the central target, respectively. The right column shows cursor trajectories in single-step trials. Each trajectory was colored with a color map that changes from red to yellow from left to right depending on the target position.

Participants (*N* = 14; mean age: 24.9 ± 1.9 years; nine male) performed the double-step trials (Fig. 1A) as the main condition and the single-step trials (Fig. 1B) as the control condition. In the double-step trials, the participants were required to launch their movements towards three potential targets, and the number of potential targets was decreased in a step-by-step manner (Fig. 1A; see “Materials and methods” for details). When the cursor went through the first threshold, participants could recognize the major direction of the potential targets by the disappearance of the right or left potential target. The condition where the center target and the left target remain at the first threshold is called the “left condition”, and the condition where the center target and the right target remain at the 1st threshold is called the “right condition”. When the cursor went through the second threshold, they could find the final target. The trial ended when 1500 ms elapsed from movement onset. A trial was considered successful if the cursor center moved through the correct target within 1500 ms after movement onset. There were two conditions of target-separation angles: a narrow (11.25º) and a wide (22.5º) condition. In the single-step trials, the final target had five possible positions, which were the same as or the middle direction of the three pre-presented potential targets (Fig. 1B; see “Materials and methods” for details). The other procedures are the same as the double-step trials. The main and control tasks were performed randomly, and participants were not able to predict the condition on each trial. The positional relationship among potential targets, cursor start position, and thresholds and trajectories from all participants in each condition are shown in Fig. 2.

**Figure 3:**
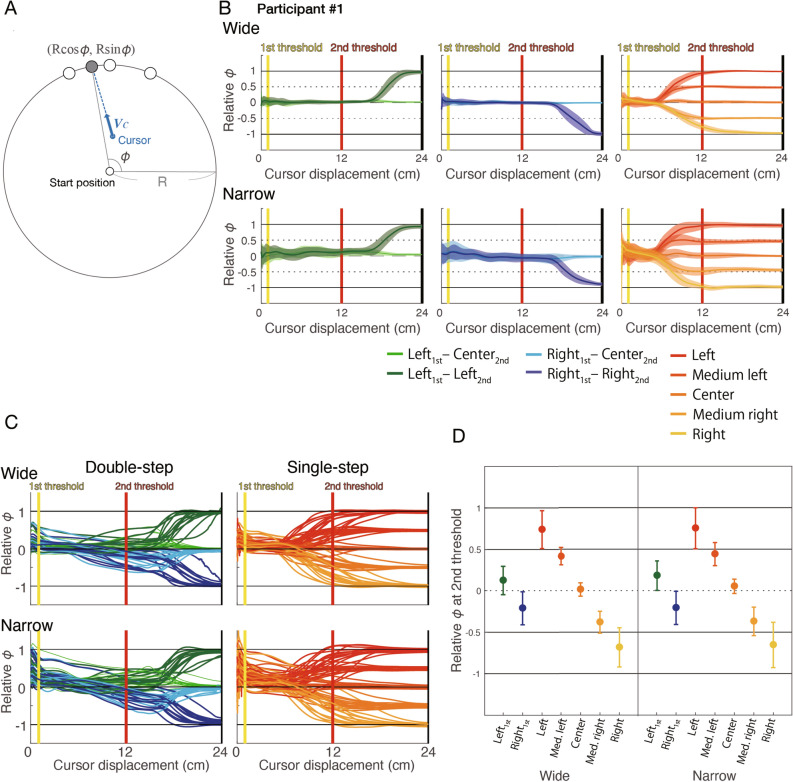
Magnitude of corrective movements depending on the cursor’s displacement. (**A**) Calculation of $\phi$. The $\phi$ angle was calculated as the angle between the horizontal vector and the vector from the start position to the estimated reach point of the vector cursor (${V}_{c}\left(t\right)$), without movement correction at each time point. This angle is an effective index to evaluate the cursor’s direction at each time point, since it is not or is barely affected by the position of the cursor, unlike the direction of the velocity vector, which is affected. (**B**) Relative $\phi$ depending on the cursor’s displacement from the start position. The relative $\phi$ was obtained by normalizing with the target-separation angles. The green and blue lines show the left and right conditions, respectively. The dark and light lines show the condition in which the final target was the side and the central target, respectively. (**C**) Mean relative $\phi$ depending on the cursor’s displacement from the start position (group data). The green and blue lines show the left and right conditions, respectively. (**D**) Mean relative $\phi$ at the second threshold between conditions (group data). Each circle indicates the between-subjects mean, and the error bars indicate the between-subjects standard deviation.

To investigate the features of motor execution dynamics over time, we calculated the cursor’s aiming direction depending on the cursor’s displacement. Several studies have used the direction of the cursor’s velocity vector^7,8^ or the direction of the cursor from the start position^3,15^ to clarify the features of motor planning towards multiple potential targets. Such indexes, however, may not be appropriate to assess the features of motor planning during movements, since the directions depend on the cursor’s position even if the cursor are moving toward the same position. In the present study, we calculated the angle ($\phi$) between the horizontal vector and the vector from the start position to the imaginary reach position of the cursor velocity vector without any movement correction (the calculation method of $\phi$ was shown in Fig. 3A), as an index of cursor direction unaffected by changes in the cursor’s position. This index indicates the cursor’s aiming position on the arc along with potential targets and is much less dependent on the cursor’s displacement than the indexes used in previous studies.

Figure 3B shows typical examples of the dynamics of relative $\phi$ normalized by the target separation angle, according to the cursor’s displacement. When the cursor proceeds towards the central potential target, the relative $\phi$ angle nears 0. When the cursor proceeds towards the left/right potential target, the relative $\phi$ angle nears 1/− 1. Thus, if the participant keeps moving the cursor towards the intermediate direction of the potential targets at each time, the relative $\phi$ angle approaches 0.5 at the level of the second threshold. In contrast, if the cursor is moved without correction after the first threshold, the relative $\phi$ nears 0 at the second threshold. This participant (#1) corrected toward the final target after the first threshold in the single-step trials, but barely changed the direction of movement before passing the second threshold in the double-step trials (Fig. 3B). Figure 3C shows the mean dynamics across participants of relative $\phi$ (normalized by the target separation angle) according to the cursor’s displacement, and Fig. 3D shows the mean relative $\phi$ between participants at the second threshold in each condition. From these figures, consistently across subjects, it is confirmed that the corrective responses between the first and second threshold in the double-step trials were small, while the correction is executed in the single-step trials.

**Figure 4:**
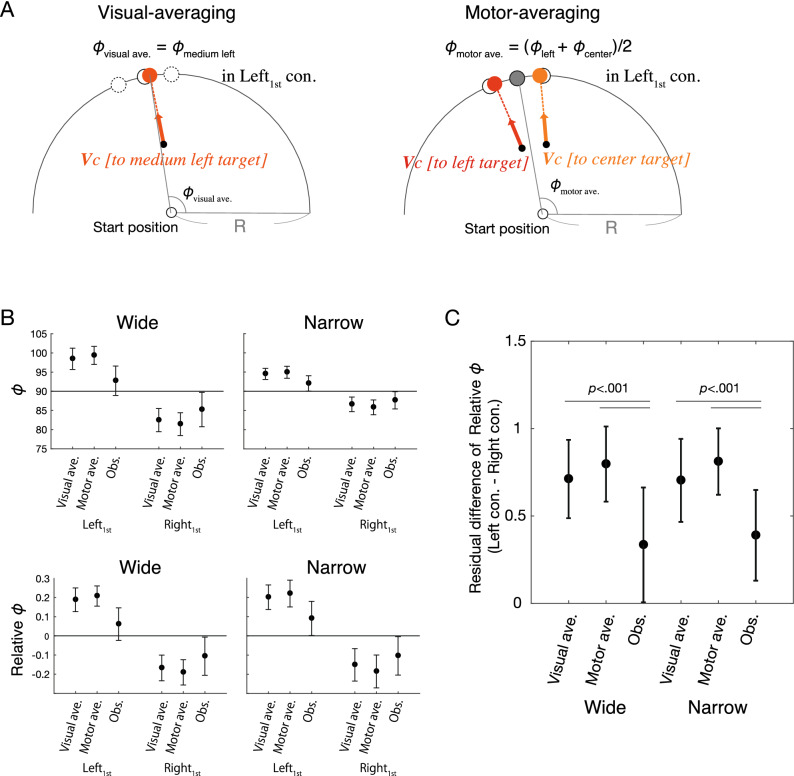
Comparison of corrective responses until the second threshold between the averaging behavior models and observed behavior. (**A**) Model assumptions. The motor-averaging model assumes that the movement direction in the-double step trials approaches the average of the single reach movements for each target in the single-step trials. For instance, the left and center targets remain at the 1st threshold, the output is the average of the movement direction when the left and center remain in the single condition. The visual-averaging model assumes that the movement direction in the double-step trials approaches the single reach movement of the intermediate target in the single-step trials. For instance, when the left and center targets remain at the first threshold, the output is the movement directions when the medium left target (i.e., the middle of the left and center targets) remains in single-step trials. (**B**) Comparison of relative $\phi$ at the second threshold among two models and observed (group data). Each circle indicates the between-subjects mean, and the error bars indicate the between-subjects standard deviation. (**C**) Comparison of the difference of relative $\phi$ values at the second threshold between remaining target directions at the first threshold (group data). A two-way repeated-measures ANOVA (3 [models] × 2 [target separation angles]) and post-hoc comparison revealed that the actual corrective responses until the second threshold were smaller than each averaging behavior model.

To identify deviations from the averaging behavior, we set up two criterion-based models of averaging behavior (i.e., visual-averaging model and motor-averaging model) and compared the estimates of these models with observed behavior. Figure 4A shows a schematic overview for each model. The visual-averaging model assumes that the participant perform a movement toward a location between the potential targets (the left panel of Fig. 4A). In other words, the participant directs the motion in the visually intermediate direction of the potential target. For example, if the left and center targets remain at the first threshold, the movement after the first threshold equivalent to moving toward the medium left target in single-step trials are expected to be taken. The motor-averaging model assumes that the average of a distinct single motor plan for each potential target is output (the right panel of Fig. 4A).

Figure 4B shows the between-participant mean of the observed relative $\phi$ values at the second threshold and those estimated by each model, and Fig. 4C shows the residual differences of these values between the left and right conditions. Two-way repeated measures ANOVA (3 [models] × 2 [target separation angles]) for the residual difference of relative $\phi$ values at the second threshold between the right and left conditions showed a significant main effect for models (*F*_[2, 26]_ = 28.96 , ${{\eta }_{p}}^{2}$ = 0.690 , *p* < 0.001). There was no significant main effect for target separation angles (*F*_[1, 13]_ = 0.416, ${{\eta }_{p}}^{2}$ = 0.031, *p* = 0.530). There was no significant interaction (*F*_[2, 26]_ = 1.342, ${{\eta }_{p}}^{2}$ = 0.094, *p* = 0.279). Post-hoc comparison revealed that there was significant differences between the observed behavior and each model (Visual-averaging vs. Observed behavior, *t*_[13]_ = 7.236, $d$ = 1.934, *p*_*bonf*_  < 0.001; Motor-averaging vs. Observed behavior, *t*_[13]_ = 5.661, $d$ = 1.513, *p*_*bonf*_ < 0.001). There was no significant difference between the models (Visual-averaging vs. Motor-averaging; *t*_[13]_ = 1.342, $d$ = 0.421, *p*_*bonf*_ = 0.382). These results suggest that the corrective responses between the 1st and 2nd threshold in double step trials deviate from the averaging behavior, and the observed corrective responses are weaker than the averaging behavior.

**Figure 5:**
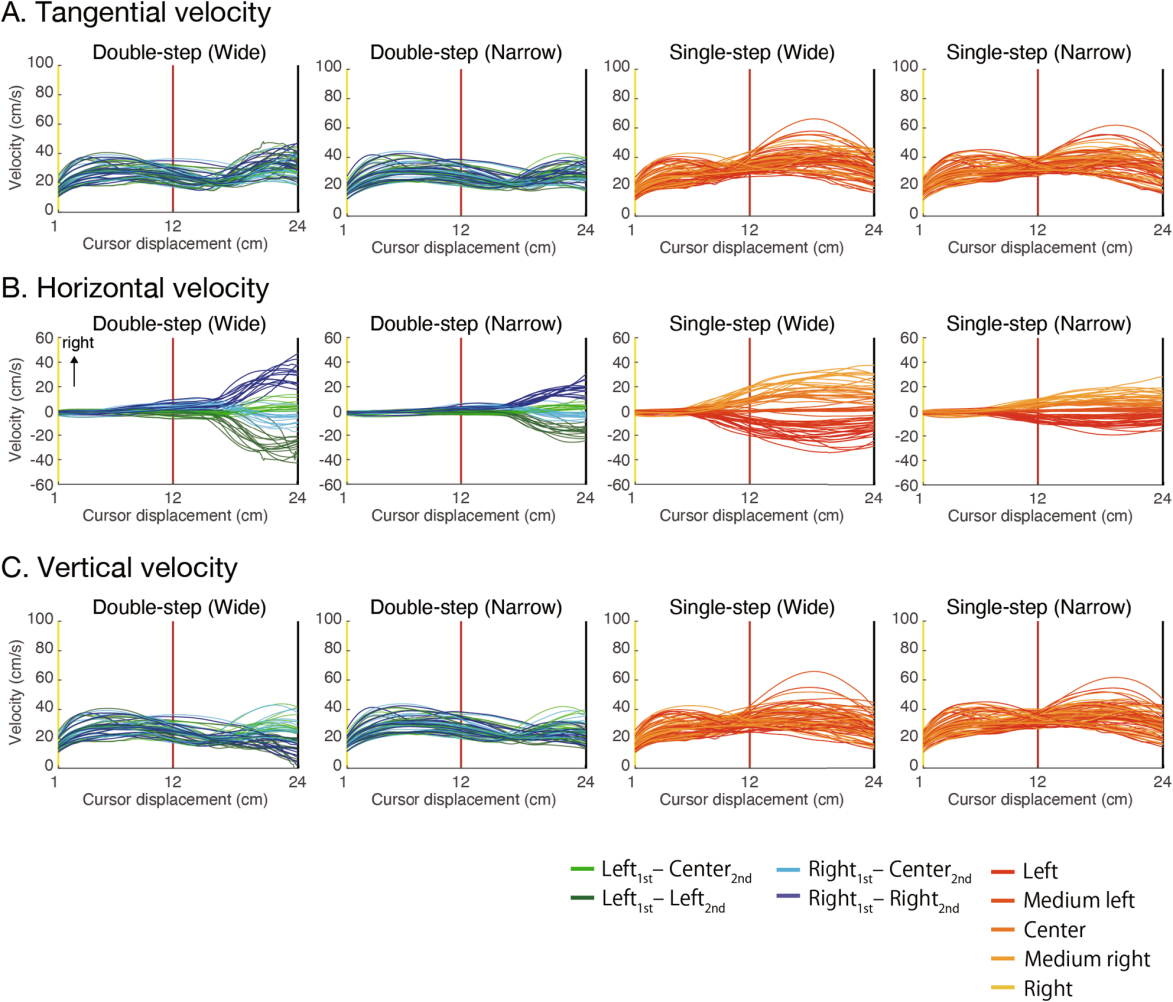
Dynamics of velocity depending on the cursor’s displacement. The top, middle, and bottom rows show the tangential, vertical, and horizontal velocities, respectively, in response to cursor movement. For the double-step trials (left and middle columns), the green and blue lines show the left and right conditions, respectively. The dark and light lines indicate the condition at which the final target was the side target and the central target, respectively. For the single-step trials (right column), the lines were colored with a color map that changes from red to yellow from left to right depending on the target position.

Besides, the velocity according to the cursor's displacement was compared between the single and double conditions. Figure 5A–C denote the tangential, lateral, and vertical velocities, respectively. From these velocity profiles, it was confirmed that the large velocity peaks appear after the final target is determined in both the single-step trials and the double-step trials. In other words, most of the participants likely took the strategy of suppressing movement correction and continuing inflight movement before the final target was determined.

## Discussion

In the current study, we investigated how humans correct their in-flight movements according to step-by-step decreases in the number of potential targets. The results showed that the participants corrected their in-flight movements less after the first threshold, compared to what was expected by the averaging behavior, including visual-averaging and motor-averaging. Besides, there were no differences in the corrective movement strategies according to the target-separation angle.

Previous studies using the go-before-you-know paradigm have reported that humans initiate their movements towards intermediate or weighted average directions between distinct motor plans toward given potential targets^1–6,10^. The weighted average behavior has been shown not only in movement paths but also in hand orientation^2,3,5,6^ and the specification of feedback gains^16^. One proposed explanation for such behaviors is the unintentional integration of movements towards each potential target^2,3,5^. If that was true in our task, the participants should have moved towards the intermediate direction between the remaining potential targets at each time-point. However, after the first threshold, they did not correct or made a weaker correction than the expected intermediate one.

Such a strategy of corrective responses may be interpreted by the optimal feedback control framework. The optimal feedback control is a computational model of movement evolution as a function of the motor system state and the way that the motor system handles variability ^11^. The control policy follows the minimum intervention principle^12–14,16–19^. A property of optimal feedback controllers is that sensed errors in state variables lead to corrections if they adversely affect motor performance but are ignored if they do not^11^. This principle was well-established on the task-dependent modulation of corrective responses to perturbations of target^20^ or cursor^21,22^. Also, this could account for the observed strategy of the corrective responses to each update of potential targets in the current study.

To correct an in-flight movement according to each reduction in the number of potential targets at the first and second thresholds can be suboptimal for the following reasons. First, the corrective response adds noise to the sensorimotor system. Second, it requires biomechanical costs. Additionally, if the participants correct their movements after both the target-revealing thresholds, multiple patterns of corrective responses should be considered. Therefore, greater cognitive efforts would be required to accomplish the task. Since humans are sensitive to costs and noise when selecting motor plans^23–30^ and feedback responses^21,31^, a reasonable strategy would be to weaken the corrective responses after the first threshold and correct their movements after the second threshold to attain the final goal.

Another possible and plausible interpretation for the weaker corrective response, compared to that estimated by the weighted average behavior, is a distortion of the subjective utility compared to the objective expected gain. In our task, from movement onset to passing over the first threshold, the probability assigned to the final goal was 25, 50, and 25% for the left, central, and right target, respectively. Therefore, in the initial phase of each trial, the central target can be assigned as the final goal in the half of the trials. Immediately after the cursor passed over the first threshold, the probability that the remaining targets (the central or the side target) were assigned as the final target was updated and became 50% for each remaining target (i.e., the probability that the side target was assigned as the final target became higher). If such an update of the probability after the first threshold is not considered, then the utility of a corrective response emphasized on the central target could be higher than that on the side target. While this notion cannot explain the difference in the magnitude of the corrective response between different target-separation angles, it can sufficiently explain the central bias of the aiming direction until the final target is revealed. In situations where the potential goals change over time, whether the potential probability or value is recognized correctly should be considered in future.

The other hypothesis was that the strategy of corrective responses would vary according to the target separation angles (i.e., wide and narrow conditions). Contrary to the previous studies showing that the setting of feedback gains is flexible according to the given target’s properties^16,21^, the current study did not show that the rates of correction varied with the target separation angles. One possible reason for this is that the target separation angels set is limited, and differences may arise when a wider target separation angle is set. Actually, a previous study with a wider range of target separation angels has shown that the emergence of averaging behavior differs depending on the target separation angle^7^.

In the current study, we tested motor planning and execution under a situation where information on the final target was revealed step-by-step, according to movement development. One of the major limitations of the current study is that we set limited task conditions, such as temporal constraints, the location of threshold, target-separation angles, and the positions of potential targets. These factors should affect the features of corrective responses during movements. In future studies, it is required to clarify how individuals modulate their motor planning/ execution according to task conditions and to model sensorimotor control policy explaining various modulations of motor planning. However, the importance of the tested situation is clear, considering that motor planning and execution in daily life occur under a continuously changing environment. The current study shows that the weighted average behavior is weakened in motor planning for ongoing movements. Although our daily motor behaviors involve sequential and overlapped planning and execution, the way that humans specify a complicated control policy for multiple perturbations according to the information on potential targets has been an open area of investigation in motor control.

In summary, the current study investigated whether humans correct their ongoing movements according to decreases in the number of potential targets in a step-by-step manner. Our results indicated that the movement direction under target revealing with step-by-step does not follow averaging behavior, and the setting of the feedback control policy to correct their ongoing movements at the final phase rather than correcting their ongoing movements at each time point. These results indicate the existence of another comprehensive optimization process, over the weighted average behavior, taking place during motor planning and execution under uncertainty about task goals.

## Materials and methods

### Participants

Fourteen right-handed, neurologically-healthy participants (age: 24.9 ± 1.9, nine male) were recruited. All participants had a normal or corrected-to-normal vision. All participants were naive to the purposes of this study and provided written informed consent. This study was approved by the Ethics Committee of the Graduate School of Arts and Sciences, the University of Tokyo. All experimental procedures adhered to the approved guidelines. Written informed consent was provided by each participant before the experiments.

### Experimental setup

The participants sat in a quiet, dim room. A pen-tablet, with sufficient workspace to measure the subjects’ arm-reach movement (Wacom, Intuos 4 Extra Large; workspace: 488 × 305 mm), was set on the table. A monitor (I-O DATA, KH2500V-ZX2; 24.5 inches, 1920 × 1080 pixels, vertical refresh rate 240 Hz) was used to present stimuli and was set with approximately 30° gradient-angle over the pen-tablet. The participants manipulated a cursor on the screen, the position of which was transformed according to the pen’s position. The elapsed time from the movement onset and the location of the cursor on the monitor were sampled at 240 Hz. All stimuli were controlled using Psychophysics Toolbox of MATLAB (MathWorks, Natick, MA, USA).

### Experimental task

The participants performed a double-step target-revealing task (double-step trials), a modified version of the typical go-before-you-know paradigm^1,3,8,15^ as the main condition, and a single-step target-revealing task (single-step trials) as the control condition. In both trials, three potential targets appeared 24 cm away from the start position at the beginning of the trial and were presented on the midline and either side of the midline at either + 11.75º and − 11.75º or + 22.5º and − 22.5º (target-separation angles between the right-sided target and the left-sided target of 22.5º and 45º, respectively). In the double-step trials (Fig. 1A), the participants were required to launch their movements towards three potential targets, and the number of potential final targets was decreased in a double-step manner. In the single-step trials (Fig. 1B), the participants were required to launch their movements towards three potential targets, and a final target was left at the first threshold.

Figure 1A shows the sequence of the double-step trials. At first, the three potential targets (white framed circles) for each trial were presented on the screen. To begin a trial, participants moved the cursor (white frame circle, radius: approximately 0.5 cm) to the start position (white frame circle, the same radius as the cursor) presented on the screen at a gradient angle of about 30° (Fig. 1Aa). If the cursor was on the start position, it turned to a filled blue circle. After a random interval (1800–2200 ms; Fig. 1Ab), an auditory beep cued participants to initiate a movement (Fig. 1Ac). The time of movement initiation was determined as the time when the cursor moved 1 cm away from the start position towards the potential targets on the screen (Fig. 1Ac). When the cursor moved past the first threshold (2 cm away from the start position), the left or right target disappeared, and the other two potential targets remained (Fig. 1Ad); at the first threshold, the central target never disappeared. The condition where the center target and the left target remain at the first threshold is called the “left condition”, and the condition where the center target and the right target remain at the 1st threshold is called the “right condition”. When the cursor moved past the second threshold (12 cm away from the start position), the true target changed to a filled red circle, and the remaining target disappeared (Fig. 1Ae). The trial ended 1500 ms after movement onset (Fig. 1Af). A trial was considered successful if the cursor center moved through the correct target. After stimuli offset, the results of the movements (“Hit” or “Miss”) and the scores (0 points for a miss trial, 100 points for a successful trial) were presented as feedback (Fig. 1Ag). The average score (i.e., the probability of a hit) was presented on the screen at the end of each set. Participants were instructed to maximize their average score.

The sequence of tasks in the single-step trials is almost the same as in the double-step trials (Fig. 1B). In the single-step trials, three potential targets were presented beforehand, and when the cursor reached the first threshold, the final target was presented. In the trials, there were five potential locations for the final target, one of the same positions as the potential targets or one of the intermediate directions of the potential targets, in order to create two averaging behavior models (details are in the “Data analysis” section). The final target location was determined in equal randomness.

In the double-step trials, there were eight patterns of the targets’ properties: two for the target-separation angles, two for the side of the remaining targets at the first threshold (left-central or right-central), and two for the final target (central or side target). In the single-step trials, there were ten patterns of the targets’ properties: two for the target-separation angles, five for the final target. In each set, five repetitions of each condition for the double-step trials and three repetitions of each condition for the single-step trials. Each set included 70 trials, and participants performed one set (70 trials) for the training session and five sets (350 trials) for the experimental session. Totally, the participants performed 420 trials. The experiment took a little over an hour.

We set the following criteria for the movement speed so that the participants could correct their movements ongoingly and smoothly. The participants were required to maintain their movement speed throughout the reach. If the movement speed slowed down by 25% of the maximum velocity in a trial until the distance of the cursor from the start position was greater than its distance from the correct target, the trial turned to miss, and the message ‘Move smoothly’ was presented on the screen as feedback. The movement velocity was controlled by the time limit of their movements (1500 ms after movement initiation).

### Data analysis

The observed data were analyzed using programs written in MATLAB. The data obtained in the last four sets, including 280 trials, were used for analysis. The cursor positions [horizontal position:$Xc\left(t\right)$, vertical position: $Yc\left(t\right)$] at each time point ($t$) were smoothed using a second-order, zero-phase-lag, low-pass Butterworth filter with a cutoff frequency of 8 Hz.

We calculated the cursor’s vector (${{\varvec{V}}}_{c}\left(t\right)=[\frac{Xc\left(t\right)}{dt}, \frac{Yc\left(t\right)}{dt}]$) at each time point ($t$). Next, we calculated the angular difference $\phi (t)={\mathrm{cos}}^{-1}(\frac{{{\varvec{V}}}_{y} \cdot {{\varvec{V}}}_{e}(t)}{{|{\varvec{V}}}_{y}||{{\varvec{V}}}_{e}(t)|})$ between the horizontal vector ${{\varvec{V}}}_{y}$ and vector ${{\varvec{V}}}_{e}\left(t\right)=\left[{x}_{e}\left(t\right), {y}_{e}\left(t\right)\right],$ where ${x}_{e}\left(t\right)$ and ${y}_{e}\left(t\right)$ are coordinates of the intersection point of the cursor vector ${{\varvec{V}}}_{c}\left(t\right)$ and the circle defined by the three potential targets, from the start position to the reach position of the cursor’s vector without movement correction, as an index indicating the features of movements at each time point (Fig. 2A). To normalize the calculated $\phi$ angle based on the cursor’s displacement, we used a cubic spline interpolation to determine $\phi$ in the interval of 1/1000 of the distance from the start position to the targets. The relative $\phi$ was normalized by the target-separation angles. If the relative $\phi$ is zero, the cursor moves towards the central target. If the relative $\phi$ is 1/− 1, the cursor moves towards the left/right target. We calculated the $\phi$ and relative $\phi$ angles at the second threshold as an index of the corrective response after the first threshold.

Two models were constructed to reflect the averaging behavior: a visual-averaging model and a motor-averaging model (Fig. 4A). The visual-averaging model assumes that the movement direction in the double-step trials approaches the single reach movement of the intermediate target in the single-step trials (in the left condition: ${\phi }_{visual\;ave.}\left(t\right)={\phi }_{single-medium\; left }\left(t\right)$, in the right condition: ${\phi }_{visual \;ave.}\left(t\right)={\phi }_{single-medium\; right }\left(t\right)$). The motor-averaging model assumes that the movement direction in the-double step trials approaches the average of the single reach movements for each target in the single-step trials (in the left condition: ${\phi }_{motor\; ave.}\left(t\right)=\frac{1}{2}{\phi }_{left \; in \; single-step }\left(t\right)+\frac{1}{2}{\phi }_{center\; in \;single-step }(t)$, in the right condition: ${\phi }_{motor \;ave.}\left(t\right)=\frac{1}{2}{\phi }_{right \; in \;single-step}\left(t\right)+\frac{1}{2}{\phi }_{center\; in\; single-step }(t)$). To test whether deviations from the averaging behavior existed, the values of the two models and the measured values at the time of passing the 2nd threshold was calculated as representative values. In order to compare the magnitude of the correction, the residual difference between the left and right conditions of the model and the observed values were calculated and compared.

### Statistical analysis

We conducted a two-way repeated-measures ANOVA (3 [two averaging models and observed behavior] × 2 [target separation angles]) for the residual difference of relative $\phi$ values at the second threshold between the right and left conditions. We conducted post-hoc comparisons using t-tests with Bonferroni correction. A partial *η*^2^ for ANOVA and Cohen’s *d* for post-hoc *t*-tests were used to report effect sizes.

## Data Availability

The data sets generated and analyzed during the current study are available from the corresponding author upon request.
